# Two cases of cryptogenic organizing pneumonia masquerading as tuberculosis (TB) in a TB endemic area

**DOI:** 10.1002/rcr2.883

**Published:** 2021-11-29

**Authors:** Mohamed Faisal Abdul Hamid, Sai Banu Selvarajah, Nik Nuratiqah, Ng Boon Hau, Andrea Yu‐Lin Ban

**Affiliations:** ^1^ Respiratory Unit University Kebangsaan Malaysia Medical Centre Kuala Lumpur Malaysia

**Keywords:** diffuse parenchymal lung disease, organizing pneumonia, tuberculosis

## Abstract

Before the era of COVID‐19 pandemic, organizing pneumonia (OP) is often underdiagnosed while tuberculosis (TB) is overdiagnosed especially in an endemic area. We report two patients with cryptogenic OP mimicking TB. First, a 56‐year‐old lady with right upper lobe air space opacity and, second, a 37‐year‐old lady with left upper lobe cavitary lesion. They were treated empirically for pulmonary TB as they had chronic cough with typical chest imaging findings. As there were no improvements despite anti‐TB and investigations for TB were negative, they underwent image‐guided biopsy which confirmed OP. Both patients received 6 months of corticosteroids therapy and made complete recovery. These cases highlight the rare presentation of OP and serves as a reminder that patients tested negative for TB, despite typical history and chest imaging findings, warrant further investigations as many diseases may mimic TB and vice versa.

## Graphical abstract

We report two patients with cryptogenic organizing pneumonia (OP) mimicking tuberculosis (TB). These cases highlight the rare presentation of OP and serves as a reminder that patients tested negative for TB, despite typical history and chest imaging findings, warrant further investigations as many diseases may mimic TB and vice versa.

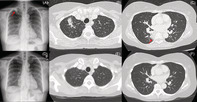

## INTRODUCTION

Organizing pneumonia (OP) was previously known as bronchiolitis obliterans with OP (BOOP). Based on its clinical, radiological and histological properties, it was then classified as an interstitial lung disease.^1^ The aetiology of OP is unknown, although there are some correlations with infectious as well as systemic diseases. Clinical features are non‐specific which include dyspnoea, prolonged cough and bilateral crackles.^2^ Histopathological examination is required to support the diagnosis. Corticosteroids are the mainstay of treatment apart from treatment of the underlying aetiology.^3^

## CASE REPORT

### Case 1

**FIGURE 1:**
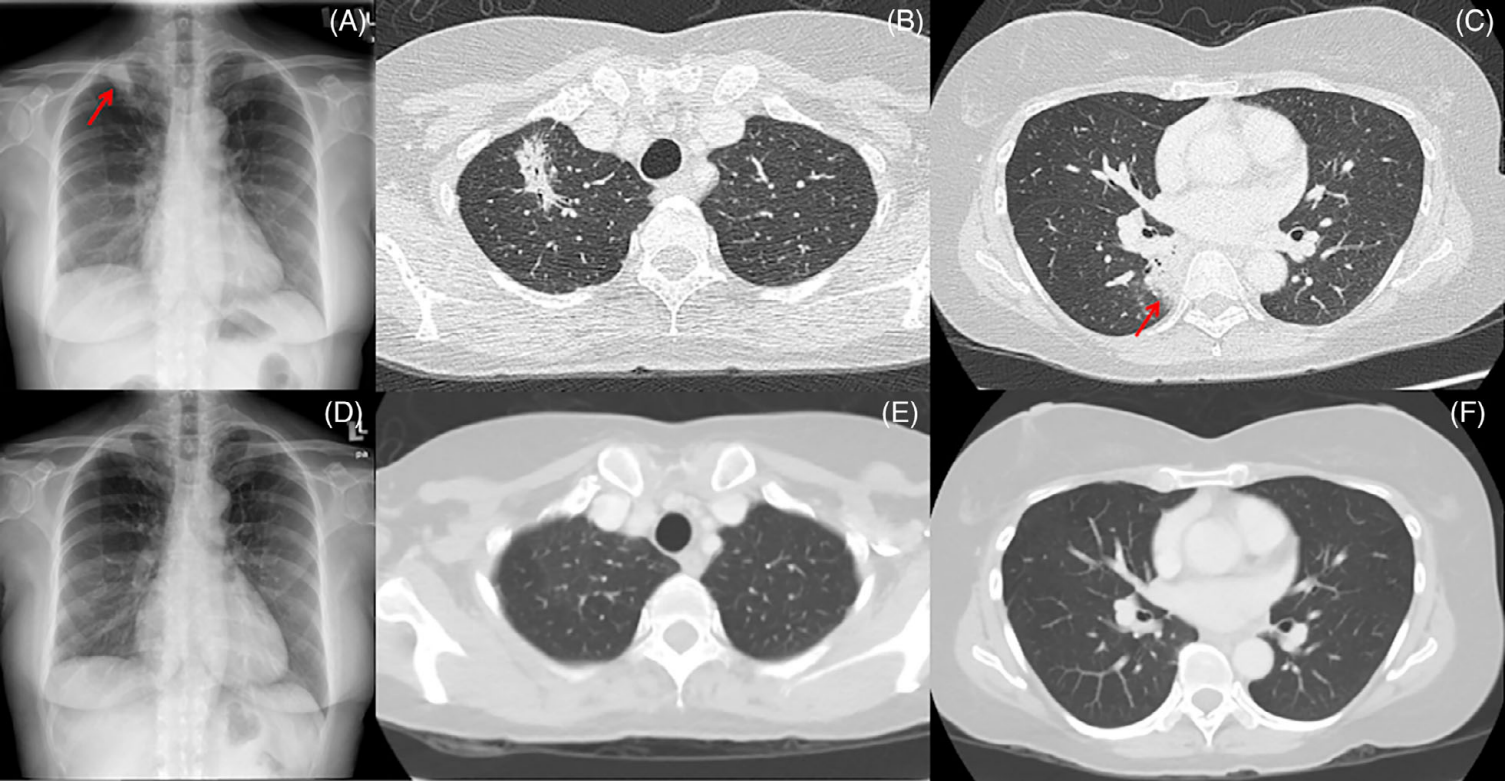
Chest radiograph (A) on initial presentation showed air space opacity seen in the right lung apex (red arrow). Axial view of the computed tomography (CT) thorax showed consolidation with air bronchogram at the right upper lobe (B) and superior segment of the right lower lobe (C) (red arrow). Chest radiograph (C) after 3 months of corticosteroids therapy revealed improvement in the right apical lung consolidation. Axial view of the CT thorax (E and F) showed resolution of previously reported multifocal lung consolidations

A previously healthy 56‐year‐old lady presented with a 4‐week history of dry cough with mild weight loss. She had no fever, haemoptysis or night sweats. There were no close contacts. Her symptoms did not resolve despite two courses of antibiotic. There were coarse crepitations at the right upper and lower zones on auscultation. Chest radiograph revealed right upper lobe air space opacity (Figure 1A). Computed tomography (CT) thorax revealed multifocal consolidations involving the right upper lobe (Figure 1B) extending to the right lower lobe (Figure 1C). She was started empirically on anti‐tuberculosis (TB) treatment while waiting for further investigations. However, after 2 weeks of treatment, there was no improvement of her symptoms. Her Mantoux tuberculin skin test (TST) showed induration of 10 mm. Sputum induction for acid‐fast bacilli (AFB) was negative, and bronchoalveolar lavage (BAL) was also negative for Xpert® *Mycobacterium tuberculosis* (MTB)/rifampicin assay as well as bacterial and fungal culture. Hence, she was subjected to CT‐guided biopsy which was consistent with the diagnosis of OP, whereby there was presence of Masson bodies and aggregates of foamy macrophages within alveolar spaces. She was screened for connective tissue disease (CTD). Her antinuclear antibody and anti‐double stranded DNA antibody tests were negative. Her diagnosis was revised as cryptogenic OP (COP) and the patient was started on prednisolone 0.5 mg/kg which was tapered over 6 months. She was asymptomatic after 1 month of treatment. Her TB culture was negative. Chest radiograph at 3 months (Figure 1D) and CT thorax (Figure 1E,F) 6 months later showed complete resolution of the consolidation.

### Case 2

**FIGURE 2:**
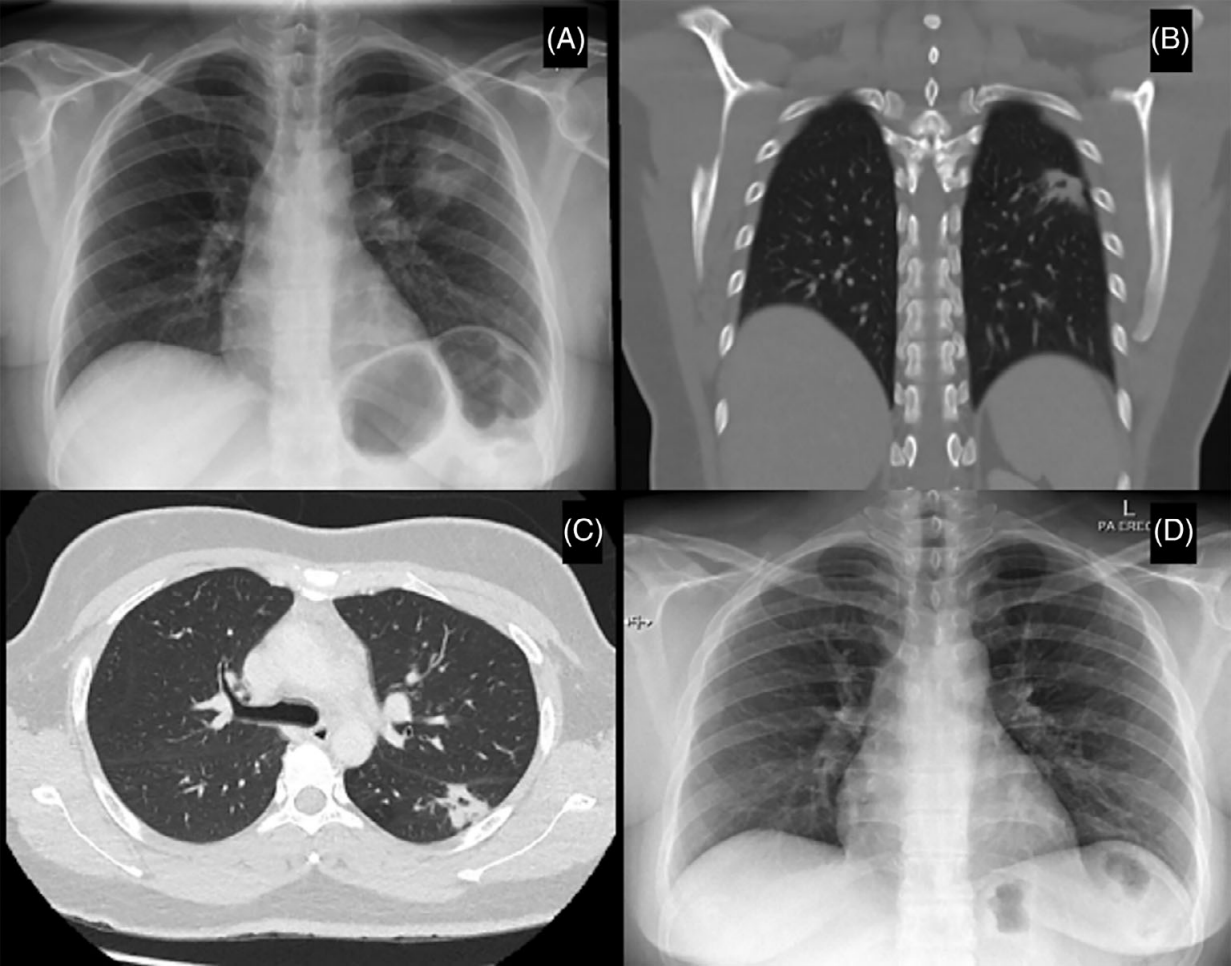
Chest radiograph (A) on initial presentation showed left midzone cavity. Coronal (B) and axial (C) views of the computed tomography thorax revealed spiculated thick‐walled cavitating lesion at the superior segment of the left lower lobe abutting the oblique fissure. Chest radiograph (D) after 6 months of corticosteroids therapy revealed resolution of the left midzone cavity

A 37‐year‐old home baker with no known medical illness presented with cough for 4 weeks with intermittent haemoptysis associated with left upper back pain and left pleuritic chest pain. There were no other symptoms such as fever, night sweats or shortness of breath. Clinically, she had clear lungs upon auscultation and no palpable lymph nodes. Spontaneous sputum AFB was negative. Erythrocyte sedimentation rate was raised at 80/h and Mantoux TST revealed 12 mm induration. Chest radiograph (Figure 2A) revealed left midzone cavity. CT thorax showed spiculated thick‐walled cavitating lesion at the left lower lobe, measuring 1.9 × 2.3 × 1.8 cm (Figure 2B,C). The patient was then treated empirically as smear‐negative pulmonary TB (PTB) and bronchoscopy was done. However, BAL also did not show any evidence of TB as the Xpert® MTB/rifampicin assay and AFB were negative. Respiratory culture from BAL was also negative. Despite undergoing anti‐TB treatment for 1 month, the patient's symptoms persisted; hence, CT‐guided lung biopsy was done which showed the presence of Masson bodies consistent with OP. CTD screening was negative. The patient was started on tapering dose of oral prednisolone at 0.5 mg/kg and follow‐up radiograph a month later showed improvement. Her TB culture after 2 months was negative. At 6 months, air space opacity on chest x‐ray had resolved (Figure 2D). As she was asymptomatic thereafter, she was discharged from our clinic.

## DISCUSSION

OP is the end product of pulmonary tissue repair process in which the aetiology can be broadly categorized as infectious (bacterial, viral, Mycobacterium and fungal) or non‐infectious. Recently, the most common cause of OP worldwide is post‐COVID infection whereby the radiological abnormalities are most severe 10 days after the initial onset of symptoms.^4^

COP is the terminology when the aetiology is unknown. COP was included in the American Thoracic Society/European Respiratory Society International Consensus Classification of the Idiopathic Interstitial Pneumonias. Both of our patients had COP. They underwent thorough investigations such as BAL to rule out infectious cause. CTDs are one of the causes of OP.^5^ In both female patients, CTD screening was also done which was negative.

With regard to initial management, anti‐TB treatment was started based on symptoms and radiology. Both patients had chronic cough. The first patient had nodular opacity on the right upper zone of her chest x‐ray and the second patient had left midzone cavity. The clinical practice of starting empirical treatment for PTB based on symptoms and chest x‐ray findings reduces time to treatment in PTB endemic region. In both patients, the radiological findings are not typical for OP. In OP, lower zone subpleural or peribronchial bilateral patchy alveolar air space consolidation is classically described on plain radiograph. On high‐resolution CT, patchy ground‐glass opacities in subpleural and/or peribronchovascular distribution (80%) and bilateral basal airspace consolidation (71%) are typical.^6^

In our patients, further investigations were carried out in view that the investigations for TB were negative including the Xpert MTB/rifampicin assay, which has a high specificity and sensitivity for the detection of MTB. We proceeded with CT‐guided biopsy that later demonstrated the presence of Masson bodies and confirmed the diagnosis of OP. During bronchoscopy, we did not send BAL for CD4:CD8 ratio as this test was not done in our centre. In OP, BAL fluid will be predominantly lymphocytes (lymphocyte percentages of up to 40% of total cells). The ratio of CD4 to CD8 cells is decreased, and the proportion of neutrophils (particularly in the early stages) and eosinophils is also frequently increased.^7^

Although OP can be a sequalae of TB as described by Kim and Kim,^8^ it was ruled out in our cases as all investigations were negative. Our cases were unique as the diagnosis of OP was not considered initially. There was also a similar report published on two patients with symptoms and signs compatible with TB, and diagnosed as OP.^9^

The treatment of OP involves managing the underlying aetiology of OP as well as suppressing the inflammation with corticosteroids. There is no consensus regarding the optimal doses of prednisolone and optimal treatment duration. The dosage is generally 0.75 mg/kg/day for 1–3 months, then 0.5 mg/kg mg/day for 3 months and then 10–20 mg/day or every other day for a total of 1 year.^10^ Every‐other‐day scheduling can be successfully used for this disorder. A shorter 6‐month course may be sufficient in certain situations. However, this duration can extend up to 12 months or even longer due to relapses. A total and permanent recovery is seen in most patients, but it is also dependent on the cause or associated systemic disorders. Anecdotally, erythromycin, inhaled triamcinolone, azathioprine, cyclosporin and cyclophosphamide have been used.^5^

The cases presented here have changed our institution's practice towards empirical anti‐TB treatment as currently we perform more aggressive investigations if the patient did not show any improvements. Although clinicians do not need to have absolute diagnostic certainty prior to initiating anti‐TB treatment, the lack of response after 2 weeks of anti‐TB treatment should prompt the clinician to investigate for an alternative diagnosis and is a reasonable timeline to suspect OP. We highlighted that OP should be considered as a differential for TB as they may mimic each other.

## CONFLICT OF INTEREST

None declared.

## ETHICS STATEMENT

The authors declare that appropriate written informed consent was obtained for the publication of this manuscript and accompanying images.
